# Cyclophosphamide exposure assessed with the biomarker phosphoramide mustard-hemoglobin in breast cancer patients: The TailorDose I study

**DOI:** 10.1038/s41598-021-81662-1

**Published:** 2021-02-01

**Authors:** S. A. M. Gernaat, H. von Stedingk, M. Hassan, H. P. Nilsson, K. A. Rodriguez-Wallberg, E. Hedayati, P. Rydberg

**Affiliations:** 1 Clinical Epidemiology Division, Department of Medicine Solna, Karolinska Institutet, Karolinska University Hospital, Stockholm, Sweden; 2 Goodpoint AB, Stockholm, Sweden; 3 Department of Laboratory Medicine, Karolinska Institutet, Karolinska University Hospital, Huddinge, Sweden; 4 Adduct Analys, Södertälje, Sweden; 5 Department of Oncology-Pathology, Karolinska Institutet, Karolinska University Hospital, Stockholm, Sweden; 6 Department of Gynecology and Reproduction, Karolinska Institutet, Karolinska University Hospital, Stockholm, Sweden; 7 Medical Unit of Breast Cancer, Sarcoma and Endocrine Tumours, Theme Cancer, Karolinska Institutet, Karolinska University Hospital, Stockholm, Sweden

**Keywords:** Breast cancer, Cancer therapy

## Abstract

Cyclophosphamide (CPA) dosing by body surface area (BSA, m^2^) has been questioned as a predictor for individual drug exposure. This study investigated phosphoramide mustard-hemoglobin (PAM-Hb, pmol g^−1^ Hb) as a biomarker of CPA exposure in 135 female breast cancer patients receiving CPA during three courses based on BSA: 500 mg/m^2^ (C500 group, n = 67) or 600 mg/m^2^ (C600 group, n = 68). The inter-individual difference was calculated for both groups by dividing the highest through the lowest PAM-Hb value of each course. The inter-occasion difference was calculated in percentage for each individual by dividing their PAM-Hb value through the group mean per course, and subsequently dividing this ratio of the latter through the previous course. A multivariable linear regression (MLR) was performed to identify factors that explained the variation of PAM-Hb. During the three courses, the inter-individual difference changed from 3.5 to 2.1 and the inter-occasion difference ranged between 13.3% and 11.9% in the C500 group. In the C600 group, the inter-individual difference changed from 2.7 to 2.9 and the inter-occasion difference ranged between 14.1% and 11.7%. The MLR including BSA, age, GFR, and albumin explained 17.1% of the variation of PAM-Hb and was significantly better then the model including only BSA. These factors should be considered when calculating the first dose of CPA for breast cancer patients.

## Introduction

Cyclophosphamide is a frequently used chemotherapy, often in combination with other chemotherapy types, for the treatment of breast cancer, malignant lymphomas, multiple myeloma, and neuroblastoma^1^. Cyclophosphamide is a pro-drug dependent on the metabolic capacity of the patient^1^. Mostly, individualized dose calculation of cyclophosphamide is based on the patient´s body surface area (BSA, m^2^) or body weight^2^. There is little evidence to validate these methods for accurate dose calculation of cyclophosphamide^3,4^.

Dosing by BSA or body weight has been questioned, as both are poor predictors for individual drug exposure^3–5^. Also, other factors should be taken into account, including genetic and environmental factors, that affect the individual variations in metabolism and clearance rate of cyclophosphamide which can vary by as much as four to tenfold^5^. Cyclophosphamide solely adjusted according to BSA is likely to result in a large proportion of under- or overdosed patients^6^. This could have an effect on the risk of breast cancer recurrence and drug-induced toxicity.

Cyclophosphamide is metabolized via hydroxylation in the liver to form the unstable precursor 4-hydroxy cyclophosphamide (4-OHCP)^7^, part of which degrades to the cytotoxic phosphoramide mustard (PAM)^8^. The PAM-Hb biomarker forms when PAM reacts with the N-terminal valine in hemoglobin (Hb). PAM-Hb is stable under physiological conditions and follows the life span of erythrocytes^9^. As such, PAM-Hb is very suitable as a biomarker since the adduct level reflects the relative area under the curve of the electrophile PAM. Also, the measurement is performed from a single blood sample that can be taken days/weeks after the administrated dose^9^. The developed technology, named TailorDose, is designed to estimate the accumulated blood level of PAM-Hb formed from the alkylating cytostatic drug cyclophosphamide^10^. The technology is based on the FI*R*E-procedure for determination of reactive electrophilic compounds by measurement of the correspondingly formed covalently bound adducts in blood^10^.

The current study was designed to investigate the variability of PAM-Hb in breast cancer patients, as a biomarker for cyclophosphamide exposure. Moreover, the correlation between PAM-Hb and other factors, including age and albumin that may have an effect on the levels of PAM-Hb, was investigated.

## Material and methods

### Study patients and design

Women diagnosed with breast cancer planning to receive adjuvant treatment with standard polychemotherapy that included cyclophosphamide were eligible to participate in the study performed at the Department of Oncology of the Karolinska University Hospital, Stockholm, Sweden. The Regional Ethics Review Board of the Karolinska Institutet in Stockholm approved this study (Dnr: 2011/1976—31/2 and 2012/809-32) and methods were performed in accordance with the relevant guidelines and regulations. All patients gave their written informed consent.

**Figure 1:**
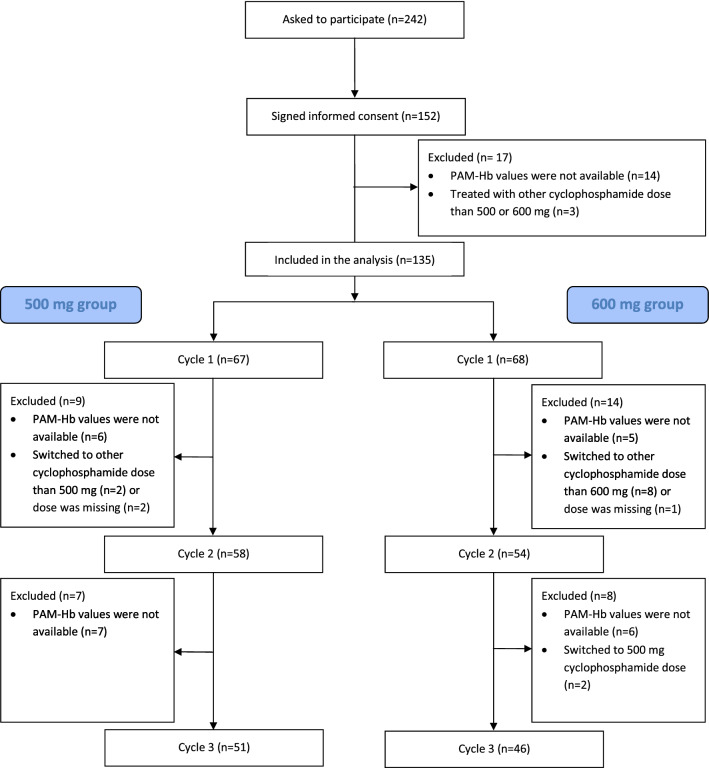
Flowchart of breast cancer patients included in the TailorDose I study, based on given dose of cyclophosphamide per body surface area during three treatment courses.

A total of 242 breast cancer patients were asked to participate in this study and 152 signed informed consent prior to enrollment during January 2012 and August 2013 (Fig. 1). The chemotherapy combination consisted of BSA adjusted doses of 5-fluorouracil, epirubicin, and cyclophosphamide (FEC) at three-week intervals. The FEC regimens were given either as three courses of F_500_E_100_C_500_ (500 mg/m^2^ 5-fluorouracil, 100 mg/m^2^ epirubicin, and 500 mg/m^2^ cyclophosphamide) or six courses of F_600_E_75_C_600_ (600 mg/m^2^ 5-fluorouracil, 75 mg/m^2^ epirubicin, and 600 mg/m^2^ cyclophosphamide). Seven patients lacked information on PAM-Hb levels for the first three courses and three were treated with other cyclophosphamide doses than C_500_ or C_600_, leaving 135 patients with data that could be analyzed. Of these, 67 patients received F_500_E_100_C_500_ and 68 patients received F_600_E_75_C_600._ The current study focused on the first three treatment courses as all patients, regardless of regimen, received these. Blood samples were withdrawn prior to the first dose course and approximately three weeks after each subsequent dose course. Blood samples were stored at − 20 °C.

### Phosphoramide mustard-hemoglobin (PAM-Hb)

The exposure of cyclophosphamide is determined via PAM-Hb, assessed from blood samples drawn after each course of each patient. An optimized and validated version of the FI*R*E procedure^9,11,12^ was used to quantify PAM-Hb, as described in detail in a previously published method validation study^9^. In short, Hb concentrations were determined in each sample before addition of the internal standard *N*-[2-(2-oxazolidonyl)ethyl]-^13^C_5_^15^*N*-valyl-leucyl-anilide (Oz-^13^C_5_^15^N-VLA, 20 pmol in 250 µL blood). Fluoresceine-5-isothiocyanate (FITC 3 mg in 30 µL DMF) was added and the samples were incubated and mixed (40 °C, 750 rpm, 8 h). Proteins were precipitated through addition of acetonitrile (1.5 ml) followed by centrifugation. Ammonium hydroxide was added to the supernatant before transfer to a solid-phase extraction mixed-mode anion-exchange cartridge for further clean-up. LC/MS in positive ion mode (ESI +), with multiple reaction monitoring (MRM), was used for the quantification of the detached N-terminal PAM-Hb adduct level.

### Covariates

The body surface area (BSA, m^2^) was calculated as √(weight (kg)* height (cm) / 3600). Smoking status during treatment was categorized into smoker and non-smoker. The body mass index (BMI) was calculated as weight (kg) divided by height (m) squared, which were measured during the first physical examination. Kidney function was investigated with the recommended glomerular filtration rate (GFR) equation from the CKD-EPI creatinine equation study using plasma creatinine levels^13^. The GFR equation was 144 × (Cr/0.7)^−0.329^ × (0.993)^age^ for women with creatinine (Cr) levels of 0.7 mg/dL and lower, and 144 × (Cr/0.7)^−1.209^ × (0.993)^age^ for women with creatinine levels over 0.7 mg/dL. Plasma creatinine and albumin levels were measured using routine measurements at the Karolinska University Hospital clinical laboratory.

### Data analyses

Patients were divided into two groups based on the first treatment course dose: (1) C500 group including patients receiving 500 mg cyclophosphamide per BSA and (2) C600 group including patients receiving 600 mg cyclophosphamide per BSA. Patients who switched to another treatment group after the first course or stopped with the treatment, were not included in the analysis from that point in time. Continuous variables with and without normally distributed data were described with means (standard deviation (SD)) and medians (interquartile range (IQR)) respectively. To examine if breast cancer patients had different PAM-Hb levels within a treatment group based on BSA, the inter-individual difference was calculated per treatment course for each group. The inter-individual difference was calculated by dividing the highest PAM-Hb value through the lowest PAM-Hb value per course. Mean PAM-Hb levels per course between the C500 and C600 groups were compared with an independent sample T-test. To examine the difference of PAM-Hb from one course to another within a breast cancer patient, the mean percentage inter-occasion difference was calculated for each individual PAM-Hb value divided by the group mean PAM-Hb per course. Subsequently, the ratio of the latter course was divided through the previous course. This calculation method was used as the clearance of cyclophosphamide differs per individual and is not equal to 100%. Crude Pearson correlations were performed to investigate the relation between PAM-Hb after the first treatment course and individual factors that may affect PAM-Hb levels including age, BSA, BMI, weight, height, GFR, height, and total given dose of cyclophosphamide after the first dose. In addition, a multivariable linear regression was performed to identify factors that explained the variation of PAM-Hb after the first treatment course of cyclophosphamide. The first multivariable linear regression model included only BSA. The second model included BSA and all parameters that showed a univariate association with PAM-Hb of *p* < 0.2 in the crude Pearson correlation^14^. Model 1 and 2 were compared with a likelihood ratio test, which compares the − 2 log likelihoods of the two models. Analyses were performed using SPSS 25.0 (SPSS Inc. Chicago, IL, USA) and SAS 9.4.

## Results

The median age at diagnosis of breast cancer patients in the C500 group was 53 years (IQR, 47 to 61), and 61 years (IQR, 49 to 68) for breast cancer patients in the C600 group, respectively (Table 1). More than half (51.4%, n = 35) of breast cancer patients in the C600 group were aged over 60 years compared to 26.9% (n = 18) in the C500 group. Compared to the C600 group, the C500 group included more breast cancer patients with histology grade III (n = 41, 59.6% vs. n = 33, 48.5%) and who smoked during treatment (n = 11, 16.4% vs. n = 7, 10.3%).

**Table 1:** Baseline demographics and characteristics of 135 breast cancer patients included in the TailorDose I study.

	500 mg cyclophosphamide	600 mg cyclophosphamide
	N	N
Number of patients—no. (%)	67 (100.0)	68 (100.0)
Hospital site—no. (%)		
Radiumhemmet, Karolinska University Hospital	53 (79.1)	56 (82.4)
Oncology clinic, Danderyd	14 (20.9)	12 (17.6)
Age at diagnosis		
Median (interquartile range), yr	53 (47–61)	61 (49–68)
Categories—no. (%)		
< 51	29 (43.3)	19 (28.0)
51–60	20 (29.8)	14 (20.6)
> 60	18 (26.9)	35 (51.4)
Weigh—kg, mean (standard deviation)	70.5 (13.3)	71.4 (12.9)
Length—cm, mean (standard deviation)	165.5 (5.0)	166.4 (6.3)
Body Mass Index—h^2^/w, mean (standard deviation)	25.7 (4.7)	25.8 (4.5)
Body surface area—m^2^, mean (standard deviation)	1.8 (0.2)	1.8 (0.2)
Primary tumour site—no. (%)		
Left	38 (57.6)	41 (60.3)
Right	28 (42.4)	27 (39.7)
Histology grade—no. (%)		
I	4 (6.0)	5 (7.4)
II	21 (29.9)	30 (44.1)
III	41 (59.6)	33 (48.5)
Not done	1 (1.5)	0 (0.0)
Estrogen receptor—no. (%)		
Positive	52 (77.6)	53 (77.9)
Negative	15 (22.4)	15 (22.1)
Progesteron receptor—no. (%)		
Positive	44 (65.7)	50 (73.5)
Negative	23 (34.3)	18 (26.5)
HER2Neu Status—no. (%)		
0	23 (34.3)	34 (50.0)
1+	15 (22.4)	14 (20.6)
2+	11 (16.4)	10 (14.7)
3+	16 (23.9)	9 (13.2)
Not done	2 (3.0)	1 (1.5)
HER2Neu Fish—no. (%)		
Yes	20 (29.9)	12 (17.7)
No	11 (16.4)	10 (14.7)
Not done	36 (53.7)	46 (67.6)
Smoking status during treatment—no. (%)		
Smoker	11 (16.4)	7 (10.3)
Non-smoker	54 (80.6)	56 (2.4)
Unknown	2 (3.0)	5 (7.3)

*HER2* Human Epidermal Growth Factor Receptor 2.

### Cyclophosphamide exposure measured with the biomarker PAM-Hb

**Figure 2:**
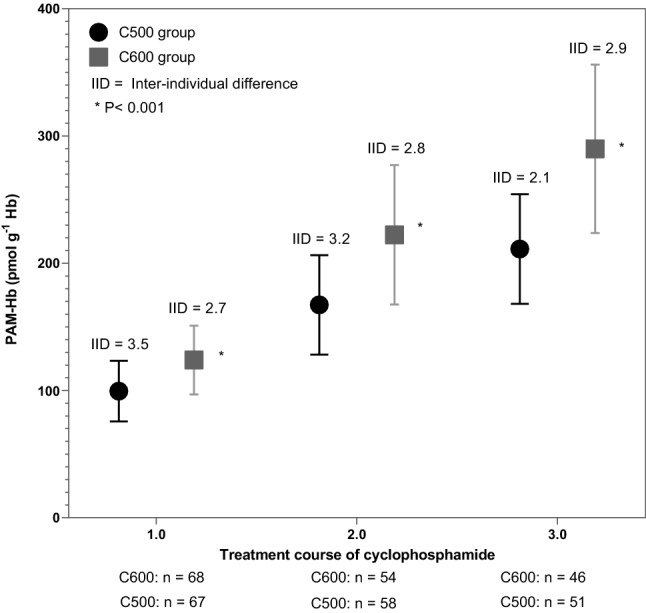
Mean (± standard deviation) levels of phosphoramide mustard-hemoglobin (PAM-Hb) for patients receiving 500 mg (C500 group) or 600 mg (C600 group) cyclophosphamide per body surface area unit during three treatment courses and the inter-individual difference per course. Difference between mean PAM-Hb levels between C500 and C600 groups was compared with an independent sample t-test. Patients were included until they changed cyclophosphamide dose or stopped the treatment.

Mean PAM-Hb levels were 99.5 pmol g^−1^ Hb for breast cancer patients in the C500 group and 124.0 pmol g^−1^ Hb for the C600 group after the first course (Fig. 2). After the third course, the mean PAM-Hb levels increased to 211.7 pmol g^−1^ Hb for breast cancer patients in the C500 group and 291.6 pmol g^−1^ Hb for breast cancer patients in the C600 group.

### Inter-individual difference of PAM-Hb

The inter-individual difference among breast cancer patients in the C500 group was 3.5 after the first course, 3.2 after the second course, and then decreased to 2.1 after the third course. The inter-individual difference was stable during the three courses in the C600 group, with 2.7 after the first course, 2.8 after the second course, and 2.9 after the third course.

### Inter-occasion difference of PAM-Hb

**Figure 3:**
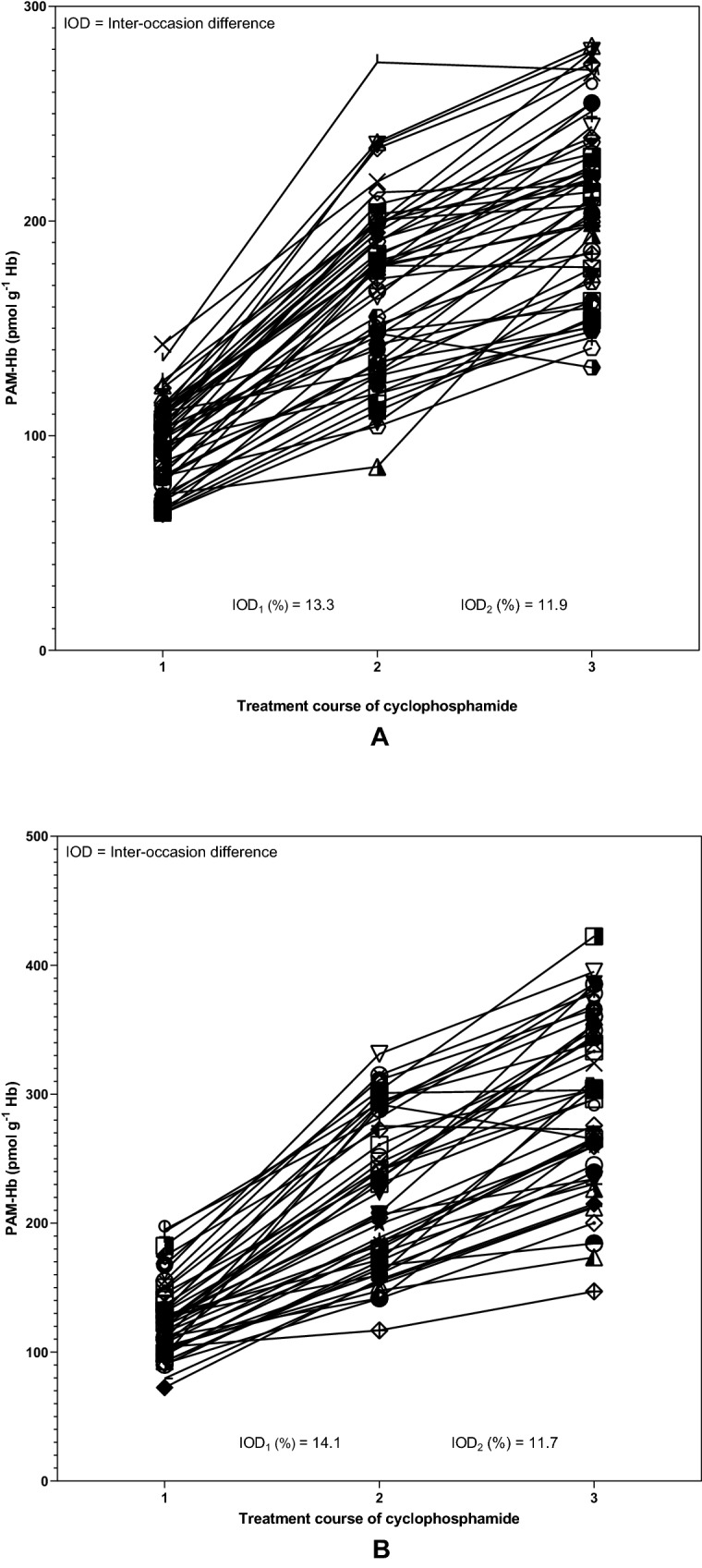
Phosphoramide mustard-hemoglobin (PAM-Hb) levels of 51 patients receiving 500 mg cyclophosphamide (C500 group) per body surface area unit (**A**) and 46 breast cancer patients receiving 600 mg cyclophosphamide (C600 group) per body surface area unit (**B**) and the mean inter-occasion difference (%) between treatment courses of cyclophosphamide.

In general, PAM-Hb levels in breast cancer patients increased after the first dose course, with a more definite increase between the first and second course than the second and third course (Fig. 3). In the C500 group (A), the mean inter-occasion difference was 13.3% between the first and second course and 11.9% between the second and third course. In the C600 group (B), the mean inter-occasion difference was 14.1% between the first and second course and 11.7% between the second and third course. Overall, the PAM-Hb levels are lower among breast cancer patients in the C500 group than the C600 group.

### Correlations with PAM-HB

**Figure 4:**
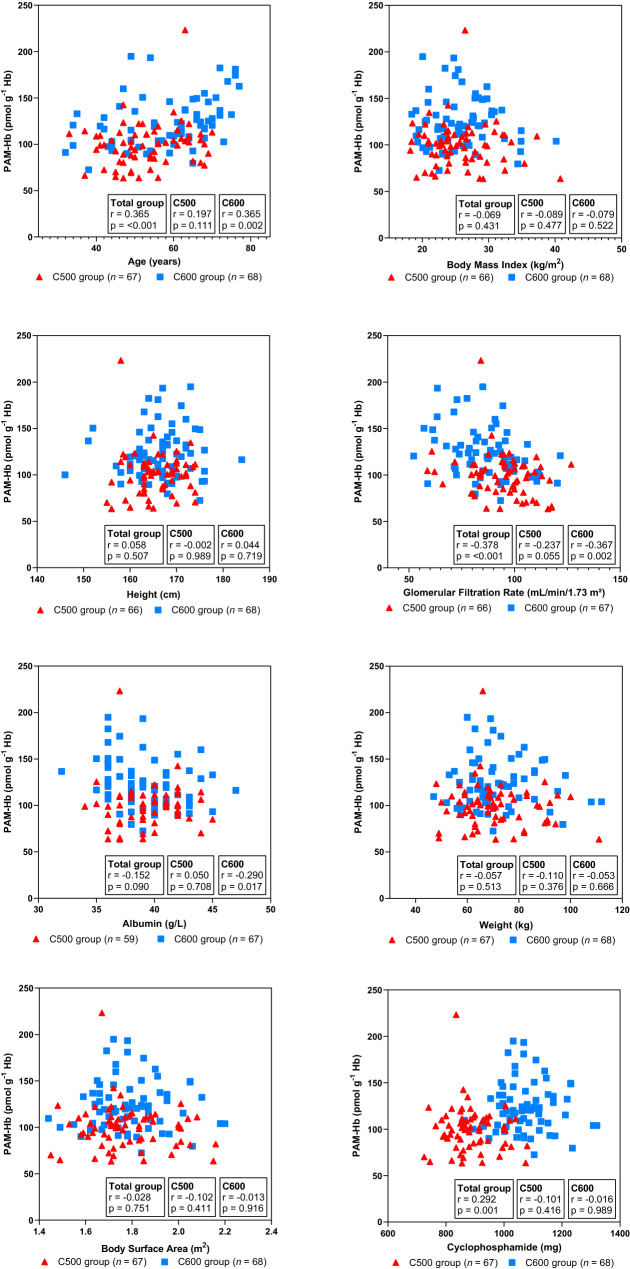
Correlations between phosphoramide mustard-hemoglobin (PAM-Hb) and parameters effecting the cyclophosphamide exposure level in 135 breast cancer patients treated with 500 mg (C500 group) or 600 mg (C600 group) cyclophosphamide per body surface area unit in the first treatment course.

After the first dose course in the total breast cancer patients, PAM-Hb was significantly correlated with age (r = 0.365, *p* < 0.001), GFR (r =  −0.378, *p* < 0.001), and total given dose of cyclophosphamide (r = 0.292, *p* = 0.001) (Fig. 4). Only in the C600 group, age (r = 0.365, *p* = 0.002), GFR (r =  −0.367, *p* = 0.002), and albumin (r =  −0.290, *p* = 0.017) were significantly correlated with PAM-Hb.

### Multivariable analysis

To explore which factors are associated with PAM-Hb after the first treatment course of cyclophosphamide and could explain the variation of PAM-Hb, a multivariable linear regression analysis was performed (Table 2). Model 1 included only BSA (β =  −0.03, *p* = 0.75), explained 0.1% of the variation with a R square of 0.001, and had a − 2log likelihood of 1280.164. Model 2 included BSA (β =  −0.81, *p* = 0.34), age (β = 1.97, *p* = 0.07), GFR (β =  −0.25, *p* = 0.02), and albumin (β =  −0.03, *p* = 0.71) as variables, explained 17.1% of the variation with a R square of 0.171, and had a − 2log likelihood of 1180.700. The likelihood ratio test showed that model 2 was significantly better than model 1, χ^2^ (degrees of freedom = 3) = 99.46, *p* < 0.001.

**Table 2:** Multivariable linear regression models including factors that are associated with PAM-Hb in breast cancer patients treated with cyclophosphamide.

Model 1		
Variable	β	*p*-value
Body surface area	− 0.03	0.75
*R* square = 0.001		

Model 2		
Variable	β	*p*-value
Body surface area	− 0.81	0.34
Age	1.97	0.07
Glomerular filtration rate	− 0.25	0.02
Albumin	− 0.03	0.71
*R* square = 0.171		

*β* standardized beta coefficient.

## Discussion

The current study showed that cyclophosphamide exposure (expressed as PAM-Hb), varied within and between courses in breast cancer patients, regardless of treatment groups based on BSA i.e. 500 mg or 600 mg. Overall, the majority of patients who started with a low or high PAM-Hb level after the first course continued with a low or high PAM-Hb level, respectively, following the second and third course. Furthermore, this study showed that age, GFR, albumin, and the total given dose of cyclophosphamide are associated with PAM-Hb. The multivariable linear regression model including BSA, age, GFR, and albumin explained 17.1% of the variation of PAM-Hb and was significantly better than the model including only BSA.

The current study used a novel analytic procedure for quantification of PAM-Hb formed from PAM, the cytotoxic metabolite of cyclophosphamide, which is robust in terms of selectivity, stability, accuracy, and precision^9^. Only a small amount of blood is needed to measure PAM-Hb (0.5 ml) and the blood sample can be taken days after cyclophosphamide has been excreted^9^. These advantageous features indicate that PAM-Hb can be used in clinical practice to examine the exposure of cyclophosphamide in patients and used in clinical studies. Previous research mainly focused on the first metabolite of cyclophosphamide, 4-HO-CPA^15,16^. However, as 4-HO-CPA is extremely unstable and occurs at low concentrations, the samples must be instantaneously processed and therefore not usable for routine clinical care.

The difference between mean PAM-Hb level after the first course in the C500 and C600 groups, i.e. 99.5 pmol g^−1^ Hb and 124.0 pmol g^−1^ Hb respectively, reflected the difference of administered dose of cyclophosphamide showing that PAM-Hb expressed the internal dose of cyclophosphamide. Overall, patients who received a higher dose of cyclophosphamide have higher PAM-Hb levels. The mean difference of PAM-Hb levels between patients in the C500 and C600 groups became even larger after the second course (167.3 pmol g^−1^ Hb and 222.3 pmol g^−1^ Hb respectively) and after the third course (211.2 pmol g^−1^ Hb and 289.9 pmol g^−1^ Hb respectively). This was expected as the PAM-Hb-adducts will follow the kinetics of the erythrocytes, which have a life span of approximately 120 days, and therefore the second and third dose of cyclophosphamide will add up until a steady-state level is reached after around 5th cycle^9^.

Between the first and third course of cyclophosphamide, the inter-individual difference ranged between 3.5 and 2.1 in the C500 group and 2.7 and 2.9 in the C600 group, respectively. A higher inter-individual difference within a treatment group based on BSA indicates that there are breast cancer patients who have different cyclophosphamide exposure levels although they received the same administered dose of cyclophosphamide. Whether the dose of cyclophosphamide needs to be adjusted to individual needs is unclear as the current study was not designed to investigate the therapeutic interval of cyclophosphamide as breast cancer outcomes were missing. The inter-individual difference increased in the C600 group over the three courses, whereas it decreased in the C500 group. As the groups are not uniform in all characteristics affecting metabolism, it may be that some patients in the C600 group have factors that decrease the elimination resulting in higher exposure levels of cyclophosphamide measured with the biomarker PAM-Hb. Higher age has been associated with a higher risk of cyclophosphamide toxicity^17^. Or, there are patients in the C500 group with characteristics enhancing the elimination of cyclophosphamide resulting in a lower exposure, such as good kidney function which have been associated with a lower risk of toxicity^17^. In the current study, the inter-occasion difference between the first and third courses ranged between 13.3% and 11.9% in the C500 group and 14.1% and 11.7% in the C600 group, respectively. The inter-occasion difference reflects the variation between two treatment courses. A lower inter-occasion difference is preferred as it suggests a certain predictability for the exposure of cyclophosphamide within the individual.

The multivariable analysis showed that the combination of BSA, age, GFR, and albumin significantly better explained the variance of PAM-Hb compared to only BSA. The explained variance of 17.1% could likely be strengthened by adding additional factors associated with the formation/elimination of PAM-Hb, such as genetic predisposition. Age was positively associated with the level of PAM-Hb, meaning that with increasing age, formation of PAM increased or elimination of PAM-Hb decreased resulting in higher internal dose of PAM, reflected in higher levels of PAM-Hb. This finding is in line with the previous reported findings showing that breast cancer patients of higher age are at a greater risk for cyclophosphamide related toxicities^17^. Kidney function, assessed with the GFR, was significantly negatively associated with PAM-Hb levels. This is in line with previous studies, showing that the level of cyclophosphamide is lower in patients with good kidney function^17^. BSA has been questioned as it seems to be a poor predictor of the elimination capacity of several anticancer drugs^5,18^. The current study shows that PAM-Hb levels are generally higher in the C600 group than in the C500 group. However, there is a large overlap in PAM-Hb levels between the two groups, which support the notion that it’s important to add additional factors to better predict and adjust the cyclophosphamide exposure in breast cancer patients.

The current study did not investigate the effect of other treatments such as epirubicin and 5-fluorouracil on PAM-Hb levels. As it is not known if these treatments interfere with the pharmacokinetics of cyclophosphamide, it may be interesting to investigate such associations in the future. In addition, it is important to investigate the association between levels of PAM-Hb and clinical outcomes in breast cancer patients. This knowledge can be used to assess if PAM-Hb can be used to further improve personalized treatment of cyclophosphamide.

In conclusion, the current study showed that the biomarker, PAM-Hb, varied considerably between breast cancer patients per course and between courses, within treatment groups based on BSA. Dose calculation of cyclophosphamide based on BSA has resulted in an inter-individual difference of 2.1–3.5. As such, there is room for improvement. Adjusting the dose of cyclophosphamide using a combination of factors, including BSA, age, GFR, and albumin has the potential to lower the inter-individual difference. This may be further lowered further by including other factors that are associated with the metabolism of cyclophosphamide such is genetic factors. Future studies may investigate the need to individualize the cyclophosphamide dose also for each subsequent course, and adjust the dose if necessary to achieve the targeted exposure of cyclophosphamide. Moreover, treatment efficacy in relation to the active cyclophosphamide dose levels during treatment courses is an important issue to be investigated.
